# Quantitatively characterizing reflexive responses to pitch perturbations

**DOI:** 10.3389/fnhum.2022.929687

**Published:** 2022-11-02

**Authors:** Elaine Kearney, Alfonso Nieto-Castañón, Riccardo Falsini, Ayoub Daliri, Elizabeth S. Heller Murray, Dante J. Smith, Frank H. Guenther

**Affiliations:** ^1^Department of Speech, Language, and Hearing Sciences, Boston University, Boston, MA, United States; ^2^The McGovern Institute for Brain Research, Massachusetts Institute of Technology, Cambridge, MA, United States; ^3^College of Health Solutions, Arizona State University, Tempe, AZ, United States; ^4^Department of Communication Sciences and Disorders, Temple University, Philadelphia, PA, United States; ^5^Gradutate Program for Neuroscience, Boston University, Boston, MA, United States; ^6^Department of Biomedical Engineering, Boston University, Boston, MA, United States; ^7^The Picower Institute for Learning and Memory, Massachusetts Institute of Technology, Cambridge, MA, United States

**Keywords:** computational modeling, motor control, speech production, pitch, auditory feedback

## Abstract

**Background:**

Reflexive pitch perturbation experiments are commonly used to investigate the neural mechanisms underlying vocal motor control. In these experiments, the fundamental frequency–the acoustic correlate of pitch–of a speech signal is shifted unexpectedly and played back to the speaker via headphones in near real-time. In response to the shift, speakers increase or decrease their fundamental frequency in the direction opposing the shift so that their perceived pitch is closer to what they intended. The goal of the current work is to develop a quantitative model of responses to reflexive perturbations that can be interpreted in terms of the physiological mechanisms underlying the response and that captures both group-mean data and individual subject responses.

**Methods:**

A model framework was established that allowed the specification of several models based on Proportional-Integral-Derivative and State-Space/Directions Into Velocities of Articulators (DIVA) model classes. The performance of 19 models was compared in fitting experimental data from two published studies. The models were evaluated in terms of their ability to capture both population-level responses and individual differences in sensorimotor control processes.

**Results:**

A three-parameter DIVA model performed best when fitting group-mean data from both studies; this model is equivalent to a single-rate state-space model and a first-order low pass filter model. The same model also provided stable estimates of parameters across samples from individual subject data and performed among the best models to differentiate between subjects. The three parameters correspond to gains in the auditory feedback controller’s response to a perceived error, the delay of this response, and the gain of the somatosensory feedback controller’s “resistance” to this correction. Excellent fits were also obtained from a four-parameter model with an additional auditory velocity error term; this model was better able to capture multi-component reflexive responses seen in some individual subjects.

**Conclusion:**

Our results demonstrate the stereotyped nature of an individual’s responses to pitch perturbations. Further, we identified a model that captures population responses to pitch perturbations and characterizes individual differences in a stable manner with parameters that relate to underlying motor control capabilities. Future work will evaluate the model in characterizing responses from individuals with communication disorders.

## Introduction

Auditory perturbation paradigms have become an important experimental approach in uncovering the neural mechanisms underlying vocal motor control. First described by Elman (1981), these paradigms involve manipulating the frequency spectrum of someone’s speech and playing it back to them via headphones in near real-time, such that they–often subconsciously–detect an error in their production. In pitch perturbation experiments specifically, the frequency spectrum is perturbed so that the fundamental frequency (*f*_*o*_; the acoustic correlate of pitch) is higher or lower than produced. In response to this manipulation, speakers will change their *f*_*o*_ in the direction opposite the perturbation, which makes what they hear in the headphones closer to what they intended to produce. When the perturbations are unexpected (for example, when applied randomly on a small percentage of trials or when applied at a random time during each trial), the compensatory response is referred to as reflexive; that is, the response is evident within a given perturbed trial but has a limited effect on subsequent trials. This contrasts with perturbations sustained over many trials that elicit both reflexive within-trial responses as well as adaptive across-trial responses (Daliri, 2021). The current work focuses on reflexive responses to pitch perturbations; we will use the term *pitch shift reflex* (PSR) to refer to such responses (Kiran and Larson, 2001).

There is a long history of utilizing reflexive responses as a diagnostic tool for probing neural function. For example, the pupillary light reflex was used by Claudius Galenus in the 2nd century to evaluate the visual capabilities of candidates for cataract surgery (see Thompson, 2003 for a historical review). Since that time, scientists have characterized the pupillary light reflex in ever-increasing detail, and modern investigations often utilize pupillography to accurately measure the time course of the pupil’s reaction to changes in light input. These studies have led to the parameterization of the temporal profile of the pupillary light reflex (e.g., Hall and Chilcott, 2018) as well as parameterized mathematical models of the dynamics of the pupillary light reflex that capture individual differences (Pamplona, 2008). The different parameters in these characterizations correspond to different neural processes; thus, an individual’s pupillary light reflex can be used to differentiate damage to one part of the nervous system from damage to another, in turn allowing clinicians to make informed decisions regarding treatment options. The dynamics of the pupillary light reflex are now used to gauge neural function in a wide range of disorders extending beyond impairment of the visual system, including concussion (Master et al., 2020), schizophrenia (Bär et al., 2008), Alzheimer’s disease (Tales et al., 2001), Parkinson’s disease (Stergiou et al., 2009), autism spectrum disorders (Fan et al., 2009), and alcoholism (Rubin, 1980). Against this background, a primary goal of the current study is to mathematically characterize the pitch reflex response using mathematical models with parameters that reflect the function of different neural subsystems involved in the control of voice.

Since the early application of the pitch perturbation paradigm, over 140 studies have used this paradigm to investigate various aspects of vocal motor control and across different populations. These studies have revealed several properties of the PSR. First, responses are typically in the direction opposite the perturbation, while a small percentage of responses occur in the same direction as the perturbation (e.g., Burnett et al., 1998; Franken et al., 2018). Second, the compensation is usually incomplete, likely reflecting an interaction between the auditory and somatosensory control systems (Smith et al., 2020). Third, the responses occur in a variety of speech stimuli (Natke and Kalveram, 2001; e.g., sustained vowels, syllables, running speech; Chen et al., 2007; Smith et al., 2020). In addition, investigations of the PSR in speakers of tonal languages, such as Mandarin, show an interaction between the linguistic intent of an utterance and perturbations, with larger responses evident when the perturbation changes the meaning of a word (Xu et al., 2004). Musicians and singers, who have higher-than-average experience controlling pitch, are also able to ignore large pitch perturbations (∼200 cents) while they compensate more completely for smaller and shorter perturbations (∼25 cents) (Zarate et al., 2010; Behroozmand et al., 2014; Parkinson et al., 2014).

While the majority of pitch-perturbation studies to date have focused on neurotypical adult speakers, a growing number of studies have examined responses in children and individuals with communication disorders. Reflexive perturbation responses in children are evident as young as age 3 years (Russo et al., 2008; Scheerer et al., 2013, 2016; Heller Murray and Stepp, 2020) but are associated with longer response latencies and greater variability compared to adult responses. Studies have also investigated responses in individuals with Parkinson’s disease (Kiran and Larson, 2001; Liu et al., 2012; Abur et al., 2021a), Alzheimer’s disease (Ranasinghe et al., 2017), cerebellar degeneration (Houde et al., 2019; Li et al., 2019), apraxia of speech (Ballard et al., 2018), aphasia (Behroozmand et al., 2018, 2022), hyperfunctional voice disorders (Abur et al., 2021b), 16p11.2 deletions (Demopoulos et al., 2018), autism (Russo et al., 2008), and in those who stutter (Loucks et al., 2012; Sares et al., 2018, 2020). Collectively, these studies shed light on the development of vocal motor control and the mechanisms underlying speech and voice disorders. In the future, these findings may inform novel treatments that directly target these mechanisms.

Compensatory responses to pitch perturbations rely on neural processes that compare the target pitch for a given utterance to the pitch as sensed through audition and apply corrections if and when an error is detected. We can use computational models to explicate these internal processes by specifying the processes with mathematical equations and evaluating how well the equations (i.e., the models) explain existing experimental data. There are several candidate model classes that may be used to model reflexive pitch perturbation data, including *Proportional-Integral-Derivative (PID), State-Space (SS), and Directions Into Velocities of Articulators (DIVA) models*.

The PID model class was originally designed to mimic the steering strategy used by expert ship helmsmen (Minorsky, 1922) and is now commonly used in a wide range of engineering applications. This model class includes proportional (P) models, where the corrective command is proportional to the error signal; proportional-derivative (PD) models, where the proportional command is supplemented with a command that is formed by multiplying the derivative of the error signal by a gain; proportional-integral (PI) models, where the proportional command is supplemented with a command formed by multiplying the integral of the error signal by a gain; and finally, PID models that combine all three error terms. SS models also originated in control engineering and have been widely applied in studies of limb motor control (Thoroughman and Shadmehr, 2000; Smith et al., 2006; Galea et al., 2015; Huberdeau et al., 2015). SS models model physical systems as a set of input, output, and state variables using first-order differential equations. The DIVA model is a prominent neural network model of speech motor control (Guenther, 2016; Kearney and Guenther, 2019). It is organized around three control subsystems, namely feedforward, auditory feedback, and somatosensory feedback control, and has been used to explain a wide number of speech phenomena. Although the SS and DIVA models have different theoretical motivations, they are closely related mathematically (as we will demonstrate) and will be treated together throughout this paper.

To the best of our knowledge, only one study to date has utilized a computational model to simulate responses to a reflexive pitch perturbation paradigm (Larson et al., 2000). Larson et al. (2000) implemented a model in which the *f*_*o*_ error was computed as the difference between the target *f*_*o*_ and actual *f*_*o*_ (following a 130 ms processing delay), partially integrated via a low-pass filter, and applied to the output. The model simulations were compared graphically to experimental data and approximated the overall timing and shape of the observed responses. The authors acknowledged that the model was likely an over-simplification of the underlying processes but nonetheless showed promise and feasibility for computational modeling of reflexive perturbation data. The current study extends this work by investigating a variety of models that utilize different numbers of free parameters to quantitatively fit pitch shift responses measured experimentally.

Both SS and DIVA models have been successfully used to simulate responses to adaptive perturbation paradigms (Daliri and Dittman, 2019; Kearney et al., 2020). Daliri and Dittman (2019) implemented an SS model with two parameters (an internal estimate forgetting factor and a sensory error weighting factor) that showed good fits to experimental data. Kearney et al. (2020) developed SimpleDIVA–a simplified version of the DIVA model–with three parameters that correspond to gains in the key subsystems involved in speech motor control (auditory feedback, somatosensory feedback, and feedforward control). SimpleDIVA also provides good fits to experimental data and is able to account for a number of variations in the sensorimotor adaptation paradigm (e.g., perturbing more than one dimension or using masking noise). An additional benefit of SimpleDIVA is that the model’s parameters provide a mechanistic explanation of behavioral responses in terms of the neural control systems believed to be involved in controlling speech production. These adaptive models, however, are not immediately applicable to reflexive response data as the mechanisms underlying the responses are not the same. Specifically, because we do not expect trial-to-trial learning in a reflexive experiment (Daliri et al., 2020; cf. Hantzsch et al., 2022), we examine the within-trial responses averaged over all perturbed trials in an experiment. Examining within-trial responses also means that we need to account for latencies associated with processing delays.

Several earlier PSR studies have observed that the compensatory response could occur on more than one time scale, resulting in a complex or multi-peaked response (Burnett et al., 1997, 1998; Larson, 1998; Hain et al., 2000). The first peak was described as a short-latency, rapid response occurring around 100-225 ms, and the second as a long-latency, slow response occurring around 250–600 ms. The simplest form of the DIVA/SS model produces only a single response peak. For this reason, we also investigate generalized versions of the DIVA/SS model that are better able to capture multi-component responses.

To address our primary goal of developing a quantitative model of the PSR, we established a model framework that allows the specification of several model variations based on PID and SS/DIVA model classes. The performance of the different models was then compared by fitting them to datasets from two prior PSR studies (Heller Murray and Stepp, 2020; Smith et al., 2020). We operationally defined model validity in terms of the ability to capture population-level responses to pitch perturbation experiments as well as individual differences in sensorimotor control processes. That is, a valid model should be able to (1) explain group mean responses to pitch perturbations, (2) have parameters that are stable across samples from an individual subject, and (3) have parameters that differentiate between individual subjects.

## Materials and methods

Our overall approach is to mathematically define a number of control models that each involve optimizable parameters. Each model generates a time series of *f*_*o*_ values, denoted by the variable *f*(*t*), where *t* ranges from 0 to the trial length of the experiment being modeled. A particle swarm optimization procedure is used to find the optimal parameter values [in terms of minimizing root-mean-square error (RMSE)] for each model when fitting a particular data set, and the resulting fit is characterized in terms of RMSE, Akaike information criterion (AIC), and cross-validated classification scores. Model fits were performed separately for two datasets from different studies involving unpredictable perturbations of *f*_*o*_ (Heller Murray and Stepp, 2020; Smith et al., 2020) applied during extended vowel productions of young healthy adult speakers.

### Datasets

**FIGURE 1:**
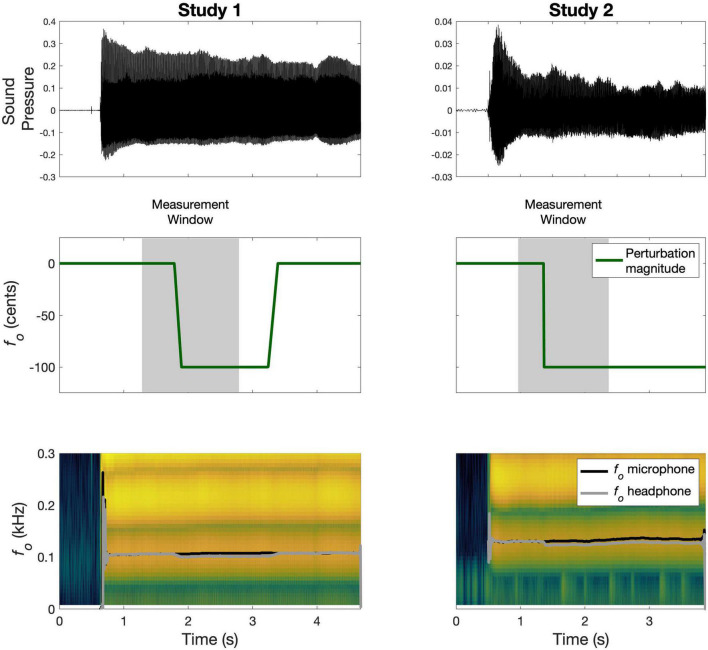
Schematic of sample perturbed trials from Study 1 and Study 2. The **top panel** shows the recorded microphone signal; the **middle panel** illustrates the time-course of the perturbation magnitude in green along with the corresponding measurement window in gray; the **bottom panel** shows the spectrogram for the microphone signal with the measured *f*_*o*_ traces overlaid in black (microphone) and gray (headphone).

In Study 1 (Smith et al., 2020), a group of English speakers (*N* = 18; aged 18–34) completed 80 trials, during which they sustained the vowel /a/ for four seconds. On a quarter (20) of all trials, an auditory perturbation of –100 cents was applied at a jittered point in time, 1,000–1,500 ms after the beginning of the trial. The perturbation was implemented as a time-domain/formant-adjusted shift using Audapter software (Cai et al., 2008); this process shifts only *f*_*o*_ while preserving the produced formants. The perturbation onset was characterized by a linear ramp that took 110 ms to reach the full perturbation magnitude. The perturbation remained on for a further 1,000–1,500 ms. The order of perturbed and control trials was pseudorandomized, with no consecutively perturbed trials. *f*_*o*_ trajectories (Hz) were extracted for the duration of the vowel using Praat (Boersma and Weenink, 2018), and then time-aligned to the beginning of the perturbation and parsed from –500 to +1500 ms in MATLAB. A schematic of a sample perturbed trial and corresponding data is shown in Figure 1. The data were normalized to the average of each subject’s baseline. On average, subjects compensated for 48.8% (*SD*: 20.8) of the perturbation, calculated as change from baseline to the last 250 ms of a trial and expressed as a percentage of the maximum perturbation magnitude.

In Study 2, a group of English speakers (*N* = 20; aged 18–28) completed 60 trials, during which they sustained the vowel /i/ for 3 s (Heller Murray and Stepp, 2020). On each trial, an auditory perturbation of +100 cents or –100 cents was applied at a jittered point in time, 500–1,000 ms after voice onset. The perturbation was implemented as a full-spectrum shift by shifting the values and spacing of the vocal harmonics using Eventide Eclipse hardware (Eventide Inc., Little Ferry, NJ, USA; Heller Murray et al., 2019), thus shifting *f*_*o*_. The perturbation onset was characterized by a step function (or sudden onset) and, once applied, the perturbation remained on for the rest of the trial. All trials in the experiment were perturbed, and the direction of the perturbation was pseudorandomized to ensure that no more than five consecutive trials were perturbed in the same direction. The intertrial interval was also jittered between 500 and 1000 ms to reduce anticipation of the next trial. *f*_*o*_ trajectories (Hz) were extracted for the duration of the vowel using Praat (Boersma and Weenink, 2018), and then time-aligned to the beginning of the perturbation and parsed from –400 to +1400 ms in MATLAB. A schematic of a sample perturbed trial and corresponding data is shown in Figure 1. To fit the models to data from both perturbation directions together, all data were normalized by dividing by each subject’s baseline average, and then flipping the upshift data around the *x*-axis. On average, subjects compensated for 17.1% (*SD*: 14.4) of the perturbation, calculated as change from baseline to the last 250 ms of a trial and expressed as a percentage of the maximum perturbation magnitude.

### Assumptions and definitions for all control models

We use the variable *f_T_* to represent the value of *f*_*o*_ that the controller is attempting to achieve; we assume this target is constant for a given speaker rather than a function of time since the experimental task being modeled involves attempting to maintain a constant pitch, and we equate *f_T_* to the average *f*_*o*_ of the speaker prior to the onset of the perturbation (i.e., during the *baseline period* between 0 and 500 ms for Study 1 and 0 to 400 ms for Study 2). Next, we assume that the output of the controlled *plant* (corresponding to the vocal tract articulators and musculature) is updated based on the signal provided by the controller at each time point^1^ as follows:


(1) $$f\left(t\right)=f_{T}+\int_{\delta =0}^{t}{\dot{f}}_{C}\left(\delta \right)d\delta$$

where *f*(*t*) is the plant output (i.e., the actual *f*_*o*_ produced by the subject) at time *t*, ${\dot{f}}_{C}\left(t\right)$ is the controller output at time *t*, and δ is a dummy variable for integration. This controller output represents a corrective command in response to the perceived error at time *t*. During the baseline period, ${\dot{f}}_{C}\left(t\right)$ is set to 0 for all models, and the baseline period is accordingly not included in RMSE calculations.

The auditory feedback of the produced sound available to the controller, corresponding approximately to the auditory cortical representation of the pitch/*f*_*o*_ of the produced sound, is defined as follows:


(2) $$f_{A}\left(t\right)=f\left(t-\tau_{A}\right)\cdot \left(1+P\left(t-\tau_{A}\right)\right)$$

where τ_*A*_ is a delay parameter that is optimized (along with other model parameters) to fit a particular dataset, and *P*(*t*) is the size of the perturbation applied at time point *t*, expressed as a percentage of *f*(*t*) in decimal form (e.g., *P* = 0.06 corresponds to a 6% upward perturbation of *f*_*o*_). The delay τ_*A*_ represents the combined delay of the perturbation processing software and hardware and the total neural processing delay from the auditory periphery to the corresponding motor output in the auditory feedback control system.

The DIVA model also includes a somatosensory representation of *f*_*o*_, assumed to derive from laryngeal mechanoreceptors, which is related to the actually produced *f*_*o*_ as follows:


(3) $$f_{S}\left(t\right)=f\left(t-\tau_{S}\right)$$

where τ_*S*_ is a delay parameter (corresponding roughly to the transmission delay from the somatosensory periphery to somatosensory cortex) that can be optimized (along with other model parameters) to fit a particular dataset. This somatosensory representation can be shown to be closely related to the parameter *A* in a typical state-space model, which weights the degree to which the current state of the system contributes to the next state (see *Basic DIVA equation* below).

### Proportional-integral-derivative equation

A PID controller is defined by the following equation:


$${\dot{f}}_{C}\left(t\right)=\alpha_{P}\cdot \left(f_{T}-f_{A}\left(t\right)\right)+\alpha_{I}$$

$$\;\cdot \int_{\delta =0}^{t}\left(f_{T}-f_{A}\left(\delta \right)\right)d\delta +\alpha_{D}$$

$$\;\cdot \frac{d}{dt}\left(f_{T}-f_{A}\left(t\right)\right)$$

which simplifies to:


(4) $${\dot{f}}_{C}\left(t\right)=\alpha_{P}\cdot \left(f_{T}-f_{A}\left(t\right)\right)+\alpha_{I}\cdot \int_{\delta =0}^{t}\left(f_{T}-f_{A}\left(\delta \right)\right)d\delta -\alpha_{D}\cdot {\dot{f}}_{A}\left(t\right)$$

where α_*P*_, α_*I*_, and α_*D*_ are optimizable gains for the position, integral, and derivative terms. We will simulate four models using this equation: a proportional model (P) in which α_*I*_ and α_*D*_ are fixed at 0, a proportional-integral (PI) model in which α_*D*_ is fixed at 0, a proportional-derivative (PD) model where α_*I*_ is fixed at 0, and a proportional-integral-derivative (PID) model in which all parameters are optimized.

### Basic directions into velocities of articulators/state-space equation

The DIVA model’s feedback controller consists of both auditory and somatosensory feedback control components. The standard formulation of the DIVA model’s feedback controller is:


(5) $${\dot{f}}_{C}\left(t\right)=\alpha_{A}\cdot \left(f_{T}-f_{A}\left(t\right)\right)+\alpha_{S}\cdot \left(f_{T}-f_{S}\left(t\right)\right)$$

where α_*A*_ and α_*S*_ are parameters denoting the gains of the auditory and somatosensory feedback control systems, respectively, and τ_*S*_ is a delay parameter corresponding to the delay between an action and the corresponding somatosensory feedback signal in somatosensory cortex. When τ_*S*_ is set to 0 [and therefore *f_S_*(*t*) = *f*(*t*); see EQ3], EQ5 is mathematically equivalent to the following SS model^2^ :


$${\dot{f}}_{C}\left(t\right)=A\cdot f_{c}\left(t\right)+B\cdot \left(f_{T}-f_{A}\left(t\right)\right)$$

where *f_c_*(*t*) = *f*(*t*) − *f*_*T*_ (see Eq. 1), B is equal to α_*A*_ in EQ5, and A is equal to -α_*S*_ in EQ5. Preliminary simulations of the two models verified this mathematical equivalence and also indicated nearly identical performance for generalized versions of the DIVA/SS models described below. The model of EQ5 is also equivalent to the low-pass filter or “leaky integrator” model proposed by Larson et al. (2000), which is a special case of EQ5 with α_*A*_ = α_*S*_ and the time constant of the low-pass filter equal to our time step size (0.005 s) times 1/α_*S*_. For simplicity, we will use the DIVA-based formulations for simulations herein as it provides a more direct physiological interpretation of model parameters than the SS or Larson et al. (2000) formulations.

### Generalized directions into velocities of articulators/state-space equations

The model of EQ5 can be generalized to include an *f*_*o*_ velocity target in addition to the *f*_*o*_ position target as follows:


$$\;{\dot{f}}_{C}\left(t\right)=\alpha_{A}\cdot \left(f_{T}-f_{A}\left(t\right)\right)+\alpha_{Av}\cdot \left({\dot{f}}_{T}-{\dot{f}}_{A}\left(t-\tau_{Av}\right)\right)+\alpha_{S}$$

$$\;\cdot \left(f_{T}-f_{S}\left(t\right)\right)+\alpha_{Sv}\cdot \left({\dot{f}}_{T}-{\dot{f}}_{S}\left(t-\tau_{Sv}\right)\right)$$

where ${\dot{f}}_{T}$ is the target velocity, α_*Av*_ and α_*Sv*_ are the auditory and somatosensory feedback control gains of the velocity-based response component, respectively, and τ_*Av*_ and τ_*Sv*_ represent the differential delays between the position and velocity components. Because subjects in the experiments being modeled were instructed to maintain a constant pitch, ${\dot{f}}_{T}$ is set to 0 and this equation reduces to:


(6) $$\;{\dot{f}}_{C}\left(t\right)=\alpha_{A}\cdot \left(f_{T}-f_{A}\left(t\right)\right)-\alpha_{Av}\cdot {\dot{f}}_{A}\left(t-\tau_{Av}\right)+\alpha_{S}$$

$$\;\cdot \left(f_{T}-f_{S}\left(t\right)\right)-\alpha_{Sv}\cdot {\dot{f}}_{S}\left(t-\tau_{Sv}\right)$$

This characterization is approximately equivalent (though not identical) to a two-state (position and velocity error) SS model.

Alternatively, the model of EQ5 can be generalized to allow two different position-error-based responses that operate at different delays:


(7) $$\;{\dot{f}}_{C}\left(t\right)=\alpha_{A}\cdot \left(f_{T}-f_{A}\left(t\right)\right)+\alpha_{As}\cdot \left(f_{T}-f_{A}\left(t-\tau_{As}\right)\right)+\alpha_{S}$$

$$\;\cdot \left(f_{T}-f\left(t\right)\right)+\alpha_{Ss}\cdot \left(f_{T}-f_{S}\left(t-\tau_{Ss}\right)\right)$$

where α_*A*_ and α_*S*_ are the auditory and somatosensory feedback control gains of the faster response component, α_*As*_ and α_*Ss*_ are the auditory and somatosensory feedback control gains of the slower response component, and τ_*As*_ and τ_*Ss*_ represent the differential delay between the fast and slow components (τ_*As*_, τ_*Ss*_ = 0). In effect, this model is a quantification of the idea that the response to a pitch perturbation includes a relatively fast, automatic component (captured by the terms involving α_*A*_ and α_*S*_) and a slower component (captured by the terms involving α_*As*_ and α_*Ss*_) that may be under more conscious control than the faster component (Burnett et al., 1997, 1998; Larson, 1998; Hain et al., 2000). This characterization is also approximately equivalent to a two-state (fast and slow position error) SS model.

### Model versions used in simulations

A total of 19 different models were tested: 4 based on PID control (models P, PI, PD, and PID) and 15 based on the DIVA model and equivalent or near-equivalent state-space formulations (D1–D15). Table 1 lists the equations and optimized parameters for all models. All unused parameters from an equation were set to 0.

**TABLE 1:** List of models included in the simulations.

Name	EQ	# Parameters	Optimized parameters
P	EQ4	2	α_P_, τ_A_
PI	EQ4	3	α_P_, α_I_, τ_A_
PD	EQ4	3	α_P_, α_D_, τ_A_
PID	EQ4	4	α_P_, α_I_, α_D_, τ_A_
D1	EQ5	3	α_A_, τ_A_, α_S_
D2	EQ5	4	α_A_, τ_A_, α_S_, τ_S_
D3	EQ6	3	α_A_, τ_*A*_, α_Av_
D4	EQ6	4	α_A_, τ_A_, α_Av_, τ_Av_
D5	EQ6	4	α_A_, τ_A_, α_S_, α_Av_
D6	EQ6	5	α_A_, τ_A_, α_S_, τ_S_, α_Av_
D7	EQ6	6	α_A_, τ_A_, α_S_, τ_S_, α_Av_, τ_Av_
D8	EQ6	6	α_A_, τ_A_, α_S_, α_Av_, τ_Av_, α_Sv_
D9	EQ6	7	α_A_, τ_A_, α_S_, τ_S_, α_Av_, τ_Av_, α_Sv_
D10	EQ6	8	α_A_, τ_A_, α_S_, τ_S_, α_Av_, τ_Av_, α_Sv_, τ_Sv_
D11	EQ7	4	α_A_, τ_A_, α_As_, τ_As_
D12	EQ7	6	α_A_, τ_A_, α_S_, τ_S_, α_As_, τ_As_
D13	EQ7	6	α_A_, τ_A_, α_S_, α_As_, τ_As_, α_Ss_
D14	EQ7	7	α_A_, τ_A_, α_S_, τ_S_, α_As_, τ_As_, α_Ss_
D15	EQ7	8	α_A_, τ_A_, α_S_, τ_S_, α_As_, τ_As_, α_Ss_, τ_Ss_

### Model parameter optimization

To fit a model to a particular dataset, a particle swarm optimization procedure was used to find optimized values of the free parameters of the model to fit a given dataset. The particle swarm optimization routine was chosen because it rapidly finds solutions in high-dimensional workspaces such as those utilized here and makes no assumptions regarding differentiability of the optimization problem. In this procedure, the system is initialized with a population of 10,000 random sets of parameter values (“particles”) and iterated until convergence to obtain an optimized parameter set. In each iteration, all parameter sets are evaluated by computing the RMSE of their fits to the data, and a fraction of all sets is replaced by random linear combinations of those parameter sets currently producing the best fits. The procedure stops when all 10,000 parameter sets converge within a 1% range of the optimal solution or after 100 consecutive iterations without any improvement in the optimal fit to the data. When the procedure stops, the optimal parameter set among the 10,000 sets from the last iteration is selected as the solution. For each model fit, the optimization procedure was run 10 times in order to evaluate any potential residual variability due to initial conditions or local optima. The resulting parameter estimates were highly robust to the initial conditions of the swarm procedure, indicative of reaching the global minimum of the RMSE measure. The minimum-RMSE solution across all 10 repetitions was chosen as the optimized parameter set, and Pearson’s *r* was calculated for this solution to characterize fit quality.

The particle swarm optimization procedure requires upper and lower bounds for the optimized parameters in order to efficiently search the parameter space. The parameter ranges for the current simulations were chosen to be big enough that they did not exclude any reasonable solutions^3^ but small enough to allow for relatively rapid convergence to the optimal solution. With this goal, the allowable range for all gain parameters was –0.1 to 1.1 with the exception of α_*I*_ in the PI and PID models, which used a range of –0.001 to 0.001 (the α_*I*_ parameter corresponds to the gain of the auditory error integral, which determines how much the corrective response increases as the error accumulates over the duration of the perturbation; preliminary simulations resulted in very tiny values for this parameter that did not always stabilize when using the larger range). A negative gain indicates a response that exacerbates, rather than corrects, the corresponding error; the negative gains allow us to model following responses. A gain of 1 corresponds to immediate full compensation for the corresponding error; gains significantly above 1 are therefore prone to instabilities and highly unlikely to represent optimal solutions. Delay parameters were limited to 0–500 ms except for the differential delays τ_*Av*_ and τ_*Sv*_, which were limited to –100 to 500 ms to allow for the possibility that the velocity error response is faster than the position error response. Preliminary simulations indicated that none of the optimized parameters were at one of the ends of the allowable range for any model; in other words, solutions were not artificially limited by the chosen bounds.

### Akaike information criterion calculations

Because adding more parameters will inevitably improve RMSE (even to the point of overfitting the data), for model comparisons we focus on AIC, which is designed to meaningfully compare models with different numbers of free parameters using the information theoretic criterion of minimum information loss^4^. The AIC for each model is defined by the equation *AIC = 2k – 2ln(L)*, where k is the number of free parameters in the model and L is the maximum likelihood of the model. We estimated the optimal model parameters for each model by minimizing the residual mean square error between the model fit and the observed traces. Assuming that the trace residuals were normally distributed but potentially correlated across timepoints, the model log-likelihood could be approximated as *ln*(*L*) = *N*/2 (−*ln*(*MSE*) − 1 − *ln*(2π)), where MSE is the mean square error of the model, and *N* is the effective degrees of freedom of the trace residuals (equal or smaller to the number of samples in the data). The degrees of freedom were computed using Satterthwaite–Welsh approximation (Satterthwaite, 1946) from the observed autocorrelation of the data before the onset of the perturbation (common to all models). Last, in order to facilitate comparisons of the resulting AIC measures across different datasets or with different studies, we reported corrected-AIC measures, dividing AIC by the data’s effective degrees of freedom, leading to the combined equation:


(8) cAIC=AIC/N=2k/N+ln(MSE)+1+ln(2π)

When comparing two models, the relative likelihood of the two models can be computed from the difference in AIC values as *exp*((*AIC*_*min*_−*AIC*)/2). To identify statistically significant differences in cAIC, we calculated the cAIC threshold necessary to support a 20:1 relative likelihood between the two models using the formula:


(9) $$thr_{cAIC}=2ln\left(1/0.05\right)/N$$

A model whose cAIC is less than another model’s cAIC by more than this threshold is, with 95% likelihood, the superior model.

### Cross-validated classification simulations

The last set of simulations further tested the models’ abilities to characterize stable properties of each subject by optimizing the models using a subset of data from each subject (training trials) and then testing the models on the remaining trials (test trials). Specifically, for each model and each subject, 10 cross-validation iterations were performed, each involving a different random subset of 10 test trials (from a total of 13–20 trials per subject in Study 1 and 19–57 trials per subject in Study 2) used for testing, with the remaining trials for that subject used as the training set for optimizing model parameters (i.e., model parameters were optimized to fit the average trace of the training trials). The optimized model was then compared to the test trials to compute a combined cAIC value, using the same cAIC formula above as in the individual-trace analyses but setting MSE to the average of the MSE values across all of the individual test trials, and setting the data samples *N* to the average effective degrees of freedom across all the test trials multiplied by the total number of trials for that model/subject combination. This led to a single cAIC value for each model and each subject, characterizing the model’s ability to predict the behavior of out-of-sample trials for an individual subject. The average of these cAIC values was then calculated across subjects for each model.

In addition, we wanted to evaluate, for each model, whether a subject’s model parameter values could be used to uniquely identify this subject’s traces from different trials compared to the traces of other subjects. The models’ abilities to correctly identify a subject were assessed from these same cross-validation iterations by first computing RMSE values comparing the average traces of one subject’s test trials to the model traces obtained from fitting the training trials of the same (or a different) subject. From these comparisons we then determined *overall* and *pairwise* classification scores for each model, from a classification procedure that chose the subject with minimal RMSE as the most likely subject to have generated that mean test trace. All classification scores represent the percent correct identifications of a subject based on the mean of 10 test trials, averaged across the 10 cross-validation iterations and all appropriate between-subject comparisons. The *overall* classification scores represent the percentage of times the correct subject (i.e., the one who generated the test trials) had the lowest RMSE when compared to all other subjects for that same model, and they were computed as:


$$p_{overall}=\frac{1}{10N}\sum_{m=1}^{N}\sum_{i=1}^{10}\prod_{n\neq m}^{N}\frac{1}{10}\sum_{j=1}^{10}\left[RMSE_{imim}<RMSE_{imjn}\right]$$

where *N* is the number of subjects, and RMSE_*i,m,j,n*_ represents the RMSE value obtained when comparing the mean trace from the test trials of the *i*-th cross-validation iteration of subject *m* to the model traces obtained from fitting the training trials of the *j*-th cross-validation iteration of subject *n*. The *overall* classification scores for each model are reported in the “Overall” columns of Table 2. Study 1 involved 18 subjects and Study 2 involved 20, so chance performance on the classification task was 5.6% for Study 1 and 5% for Study 2.

**TABLE 2:** Fit statistics for all simulated models.

	Study 1 group		Study 2 group		Study 1 subject		Study 2 subject		Study 1 Xval classification			Study 2 Xval classification		
Model	RMSE	cAIC	RMSE	cAIC	RMSE	cAIC	RMSE	cAIC	cAIC	Overall	Pair	cAIC	Overall	Pair
P	0.00312	–6.33001	0.00198	–7.1133	0.00381	–6.1104	0.00237	–5.4655	–1.6882	23.39%	84.23%	–1.3595	18.25%	72.11%
PI	0.00083	–6.41743	0.00073	–7.1847	0.00201	–6.1983	0.00130	–5.4930	–1.7201	35.94%	88.10%	–1.3708	24.14%	74.59%
PD	0.00312	–6.32608	0.00197	–7.1110	0.00355	–6.1208	0.00234	–5.4644	–1.6870	26.82%	85.53%	–1.3587	18.21%	72.11%
PID	0.00065	–6.41588	0.00071	–7.1820	0.00158	–6.2102	0.00120	–5.4926	–1.7206	38.76%	88.77%	–1.3701	24.16%	74.58%
D1	0.00059	–**6.42106**	0.00039	–**7.1932**	0.00187	–6.2049	0.00112	–5.4950	–1.7228	35.70%	88.15%	–1.3713	22.69%	73.66%
D2	0.00059	–6.41672	0.00039	–7.1901	0.00174	–6.2023	0.00100	–5.4960	–1.7187	35.72%	88.28%	–**1.3715**	24.72%	74.68%
D3	0.00312	–6.32608	0.00197	–7.1110	0.00355	–6.1208	0.00234	–5.4644	–1.6870	26.82%	85.53%	–1.3587	18.21%	72.11%
D4	0.00116	–6.40619	0.00083	–7.1780	0.00183	–6.1964	0.00145	–5.4848	–1.7157	37.75%	88.42%	–1.3669	22.04%	73.25%
D5	0.00053	–6.41737	0.00026	–7.1921	0.00151	–6.2129	0.00105	–5.4941	–**1.7233**	38.55%	88.74%	–1.3706	23.07%	73.78%
D6	0.00052	–6.41321	0.00021	–7.1895	0.00137	–6.2106	0.00092	–5.4951	–1.7195	38.73%	88.89%	–1.3708	25.56%	74.83%
D7	0.00046	–6.40946	0.00017	–7.1867	0.00113	–6.2103	0.00081	–5.4942	–1.7147	39.49%	89.00%	–1.3697	25.65%	74.93%
D8	0.00052	–6.40886	0.00017	–7.1866	0.00117	–6.2093	0.00092	–5.4918	–1.7142	38.53%	88.78%	–1.3688	23.70%	73.94%
D9	0.00052	–6.40451	0.00017	–7.1835	0.00103	–6.2065	0.00076	–5.4930	–1.7110	40.20%	89.18%	–1.3689	26.05%	75.06%
D10	0.00048	–6.40055	0.00013	–7.1806	0.00100	–6.2014	0.00071	–5.4922	–1.7070	40.37%	89.14%	–1.3679	26.12%	75.13%
D11	0.00104	–6.40902	0.00040	–7.1900	0.00147	–**6.2129**	0.00097	–**5.4964**	–1.7227	38.57%	88.93%	–1.3713	25.38%	75.08%
D12	0.00051	–6.40890	0.00027	–7.1857	0.00111	–6.2110	0.00078	–5.4948	–1.7129	39.06%	88.94%	–1.3699	25.70%	75.24%
D13	0.00051	–6.40889	0.00027	–7.1857	0.00116	–6.2099	0.00082	–5.4944	–1.7160	39.92%	89.03%	–1.3698	25.07%	75.15%
D14	0.00051	–6.40454	0.00013	–7.1838	0.00105	–6.2064	0.00076	–5.4933	–1.7110	**40.71%**	89.18%	–1.3690	25.01%	75.16%
D15	0.00035	–6.40165	0.00012	–7.1807	0.00093	–6.2027	0.00066	–5.4924	–1.7058	40.57%	**89.22%**	–1.3682	**26.32%**	**75.27%**



Xval, cross-validation; RMSE, root-mean-square error; cAIC, corrected Akaike information criterion; Overall, overall accuracy (%); Pair, pairwise accuracy (%).

Boldface type indicates the model with lowest RMSE and cAIC for each study.

Blue shading indicates cAIC values that are within the cAIC threshold of the lowest cAIC value.

*Pairwise* classification scores represent the percentage of times the correct subject had lower RMSE than another (randomly selected) incorrect subject for that same model, and they were computed as:


$$\;p_{pairwise}=\frac{1}{10N\left(N-1\right)}\sum_{m=1}^{N}\sum_{i=1}^{10}\sum_{n\neq m}^{N}\frac{1}{10}\sum_{j=1}^{10}$$

$$\;\left[RMSE_{imim}<RMSE_{imjn}\right]$$

Classification accuracies for a given model were averaged across all pairs of subjects to obtain the scores listed in the “Pairwise” columns of Table 2; chance performance on this classification task is 50%.

Additionally, intraclass correlation coefficients (ICC) were calculated to quantify the reliability/stability of model parameters across the 10 cross-validation iterations. ICC values were calculated as:


ICC=σbetween2σbetween2+σwithin2

where σ_*between*_ is the between-subject standard deviation for a given parameter and σ_*within*_ is the within-subject standard deviation for a given parameter. ICC values fall between 0 and 1, with values < .5 indicating poor reliability, values 0.5–0.75, 0.75–9, and 0.9–1 indicating moderate, good, and excellent reliability, respectively (Koo and Li, 2016).

## Results

Table 2 summarizes the fit statistics for all models and simulations. The following subsections describe these results by simulation set: fits to study group means, fits to individual subject means, and cross-validated classification simulations.

### Fits to group means

**FIGURE 2:**
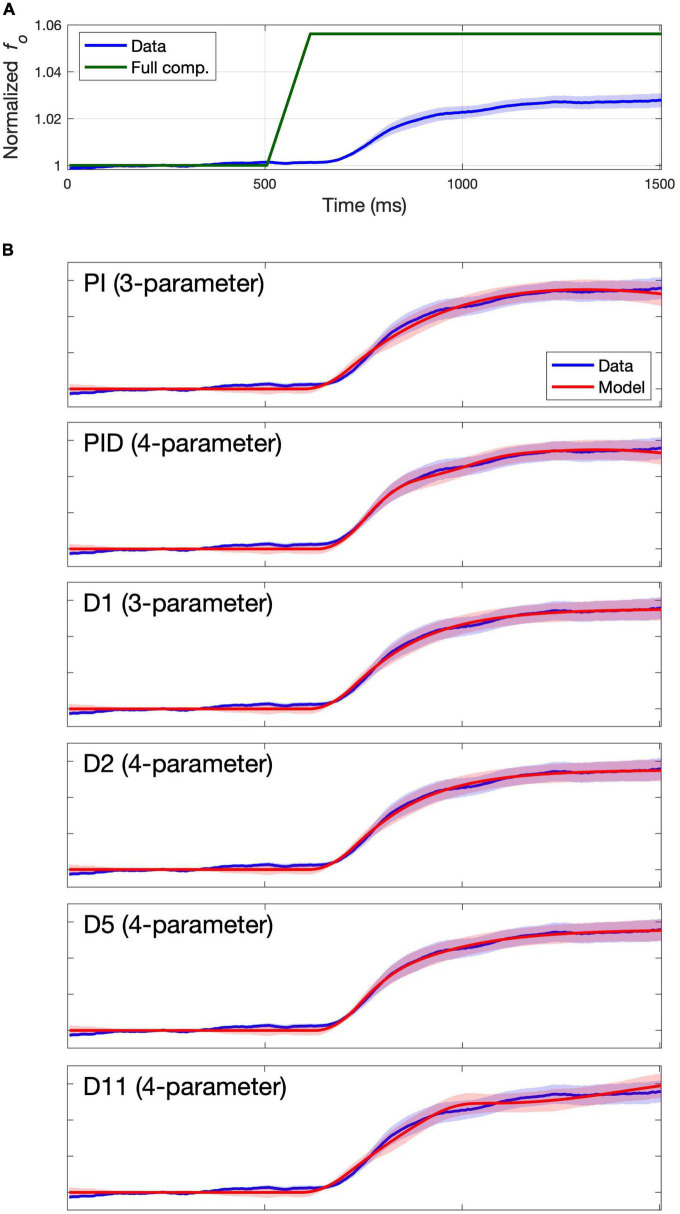
Group mean data and model fits for Study 1. Group mean data and standard error of the mean are shown with a blue line and shading. **(A)** Group mean data shown relative to full compensation in green. Full compensation is the inverse of the perturbation magnitude and illustrates what 100% compensation would look like. **(B)** Group mean data shown relative to model fit (red line) and standard error of the model fit (red shading) for models providing best fits to the group mean data.

**FIGURE 3:**
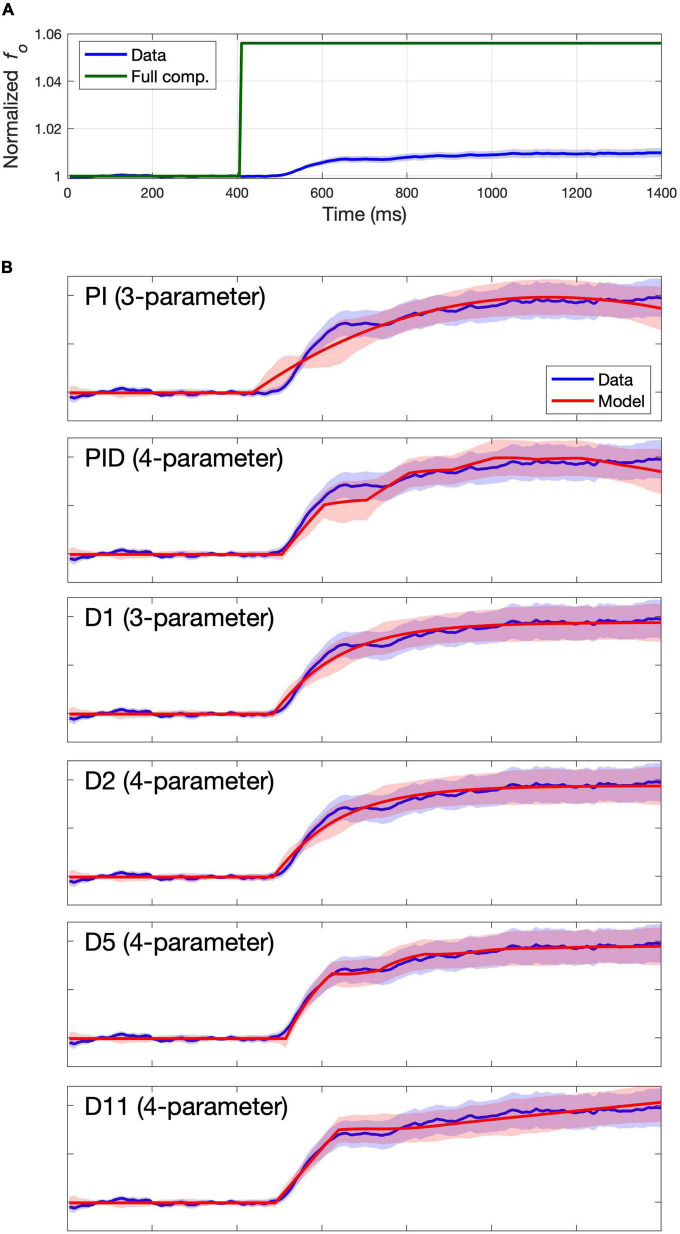
Group mean data and model fits for Study 2. Group mean data and standard error of the mean are shown with a blue line and shading. **(A)** Group mean data shown relative to full compensation in green. Full compensation is the inverse of the perturbation magnitude and illustrates what 100% compensation would look like. **(B)** Group mean data shown relative to model fit (red line) and standard error of the model fit (red shading) for models providing best fits to the group mean data.

The group mean trace for each study was formed by first calculating the mean *f*_*o*_ value at every time point for each individual subject (averaged across all that subject’s trials), then averaging these individual subject means to form the group mean trace. The group mean traces are indicated by the solid blue lines in Figure 2A (Study 1) and Figure 3A (Study 2), with standard error of the mean (SEM) indicated by blue shading. Full compensation, the inverse of the perturbation magnitude, is shown in green. Full compensation illustrates what a 100% compensation for the perturbation would look like, although this is rarely achieved in auditory perturbation studies.

The columns labeled “Group” in Table 2 indicate the RMSE for each model’s fit to the group mean trace as well as the cAIC value resulting from comparing the model fit to the individual subject mean traces. The lowest RMSE and cAIC values for each study are indicated in boldface. Blue shading indicates cAIC values that are within the cAIC threshold of the lowest cAIC value; in other words, the models with no shading are inferior to the best (boldfaced) model according to the cAIC criterion, whereas the models with blue shading are not significantly different (at the *p* < 0.05 false positive level) from the best model. For both studies, the three-parameter model D1 provided the best fit according to cAIC, with the three-parameter model PI also falling within the cAIC threshold, along with several four-parameter models (PID, D2, D5, and D11), a five-parameter model (D6), and several six-parameter models (D7, D8, D11, D12, and D13). For the remainder of this article, we will refer to models within the cAIC threshold of the best model collectively as the “best models.”

When multiple models fall within the AIC threshold of the top model, there is not enough empirical evidence to support the selection of an individual model among them. In these cases, and until more evidence becomes available, it is reasonable to give preference to the model with the fewest parameters amongst these models. Thus, according to the cAIC criterion, the models providing the best fits to the group mean data are the three-parameter models D3 and PI, followed by the 4-parameter models PID, D2, D5, and D11. Figures 2B, 3B plot the model fit (red line) and standard error of the model fit (red shading), along with the group mean (blue line) and SEM (blue shading) of the experimental data for these models. (Model fits for all models are provided in the Supplementary materials). Despite falling within the cAIC threshold of the best model D1, the PI and PID models produce traces that poorly match the overall shape of the data trace, casting doubt as to whether they are effectively capturing the physiological mechanisms responsible for the subjects’ productions. This is particularly clear from the Study 2 fits in Figure 3. The four-parameter model D5 appears to best capture the overall shape of the data trace, which shows an initial plateau followed by a second rise approximately 100 ms after the start of the plateau (again more clearly visible in Figure 3). As noted in the *Introduction*, a two-component response pattern has been noted in prior pitch perturbation experiments (e.g., Larson, 1998; Hain et al., 2000). Models D1, D2, and D11 capture the overall shape of the data trace reasonably well, but they fail to properly capture the shape of the plateau and second rise.

In sum, the three-parameter model D1 provides the best fit of the group mean traces according to the cAIC criterion while also capturing the overall shape of the experimentally measured response reasonably well. The four-parameter model D5 best captures the overall shape of the data traces amongst the three- and four-parameter models and falls within the cAIC threshold of model D1. The additional parameters of models with more than four parameters appear to provide little additional improvement.

The optimized values of all parameters for all models are provided in the Supplementary materials. For the basic DIVA best models (D1 and D2), the parameter values were very similar between models for a given dataset. For Study 1, the mean values (across the two models) were 0.011 for α_*A*_, 0.013 for α_*S*_, 115 ms for τ_*A*_, and 130 ms for τ_*S*_. For Study 2, they were 0.006 for α_*A*_, 0.033 for α_*S*_, 93 ms for τ_*A*_, and 54 ms for τ_*S*_. These parameters had similar values in the generalized DIVA best models (D5–D8 and D11–D13), whereas the additional parameters in the generalized DIVA models were considerably more variable across models.

### Fits to individual subjects

The second set of simulations compared the models on their ability to fit individual subject data using parameters optimized for the individual subject rather than the group mean. These simulations gauge how well the models can account for individual differences through subject-specific parameterizations. For each subject, model parameters were optimized to fit the subject’s mean trace (averaged across trials). The RMSE values of these fits are provided in the columns labeled “Subject” in Table 2, along with the cAIC values resulting from comparing the models’ fits to the individual subject mean traces. With the exception of models P, PD, D3, and D4, all models fell within the cAIC threshold of the best model (D11 for both studies).

### Cross-validated classification simulations

The columns labeled “Xval Classification” in Table 2 provide cAIC, overall classification accuracy, and pairwise classification accuracy for each model in each study. The models within the cAIC threshold of the best cAIC value for both studies were models PI, PID, D1, D2, D5–D7, and D11–D13. The highest overall classification accuracies were 40.71% for model D14 in Study 1 (chance level of 5.6%) and 26.32% for model D15 in Study 2 (chance level 5%). Even the worst-performing models had overall accuracies that were well above chance: 23.39% for model P in Study 1 and 18.21% for models PD and D3 in Study 2. Pairwise classification accuracies were also well above chance (50%) for all models, ranging from 84.23% (model D1) to 89.22% (model D15) for Study 1, and from 72.11% (models P and D1) to 75.27% (model D15) in study 2.

Overall, these results indicate that reflexive responses to *f*_*o*_ perturbations are largely individual-specific, and a number of models perform nearly equivalently on the cross-validated classification tasks. For comparison, we also calculated cross-validated classification accuracy when we used the mean of the training trials for classification rather than one of the models. This resulted in overall and pairwise accuracies of 38.14 and 89.25%, respectively, for Study 1 and 25.89 and 75.38% for Study 2. These are similar to values obtained for the best-performing models in Table 2.

The cross-validation training iterations also provide information regarding the stability of model parameters across the 10 iterations for a given subject. In other words, do the 10 iterations yield approximately the same values for a given parameter (as would be expected if the parameter has a reliable physiological basis) or do they vary substantially across iterations (indicative of a model whose parameters do not have a reliable physiological interpretation)? To assess this, we calculated ICC for each parameter in each model for each data set. The mean parameter values and ICC values from the 10 cross-validation iterations are provide in Table 3 (Study 1) and Table 4 (Study 2). Boldface type indicates the model with the highest ICC value per parameter. Dark blue shading indicates ICC values greater than 0.75 (corresponding to good reliability), and light blue shading indicates ICC values between 0.5 and 0.75 (moderate reliability).

**TABLE 3:** Study 1 mean values and ICC of optimized parameters in cross-validation simulations.

	α_P_/α_A_		α_S_		α_D_/α_A*v*_/α_A*s*_		α_I_/α_S*v*_/α_S*s*_		τ_A_		τ_S_		τ_A*v*_/τ_A*s*_		τ_S*v*_/τ_S*s*_	
Model	Mean	ICC	Mean	ICC	Mean	ICC	Mean	ICC	Mean	ICC	Mean	ICC	Mean	ICC	Mean	ICC
P	0.005	**0.956**							0.044	0.647						
PI	0.009	0.939					–4.9E-05	0.826	0.109	0.660						
PD	0.010	0.924			0.923	0.592			0.109	0.601							
PID	0.013	0.918			0.551	0.414	–1.0E-04	**0.842**	0.135	0.594						
D1	0.012	0.851	0.016	0.705					0.128	0.586						
D2	0.010	0.870	0.013	0.657					0.111	0.556	0.159	**0.470**				
D3	0.010	0.924			0.923	0.592			0.109	0.601						
D4	0.008	0.941			0.788	0.688			0.101	**0.690**			0.274	**0.582**		
D5	0.015	0.852	0.018	**0.772**	0.393	0.328			0.143	0.536						
D6	0.012	0.903	0.014	0.747	0.568	0.335			0.128	0.568	0.226	0.337				
D7	0.011	0.851	0.009	0.650	0.731	0.662			0.118	0.534	0.203	0.369	0.162	0.455		
D8	0.014	0.827	0.011	0.699	0.789	0.468	0.170	0.317	0.123	0.502			0.218	0.469		
D9	0.012	0.823	0.008	0.576	0.648	0.565	0.403	0.412	0.124	0.500	0.152	0.419	0.240	0.350		
D10	0.012	0.836	0.008	0.619	0.734	0.644	0.444	0.296	0.123	0.488	0.143	0.318	0.201	0.257	0.122	**0.268**
D11	0.013	0.662			–0.010	0.584			0.120	0.613			0.296	0.510		
D12	0.018	0.545	0.069	0.657	0.013	**0.694**			0.110	0.397	0.080	0.235	0.307	0.358		
D13	0.033	0.592	0.123	0.762	0.020	0.686	0.573	0.206	0.120	0.354			0.300	0.396		
D14	0.022	0.448	0.070	0.611	0.014	0.575	0.618	0.472	0.119	0.391	0.078	0.226	0.319	0.309		
D15	0.015	0.666	0.035	0.555	0.011	0.607	0.697	0.440	0.114	0.380	0.052	0.277	0.287	0.301	0.185	0.264



Only one parameter listed per column is optimized in a given model. For example, the PID models have an α_**P**_ parameter whereas the DIVA models have an α_**A**_ parameter. See Table 1 for a complete list of parameters included in each model.

ICC, intraclass correlation coefficient.

Boldface type indicates the model with the highest ICC value per parameter.

Light blue shading indicates ICC values are between 0.5 and 0.75 (moderate reliability).

Dark blue shading indicates ICC values are > 0.75 (good-excellent reliability).

**TABLE 4:** Study 2 mean values and ICC of optimized parameters in cross-validation simulations.

	α_P_/α_A_		α_S_		α_D_/α_A*v*_/α_A*s*_		α_I_/α_S*v*_/α_S*s*_		τ_A_		τ_S_		τ_A*v*_/τ_A*s*_		τ_S*v*_/τ_S*s*_	
Model	Mean	ICC	Mean	ICC	Mean	ICC	Mean	ICC	Mean	ICC	Mean	ICC	Mean	ICC	Mean	ICC
P	0.001	0.969							0.031	0.761						
PI	0.003	**0.972**					–2.6E-05	**0.955**	0.062	0.810						
PD	0.003	0.969			1.019	0.597			0.055	0.793						
PID	0.005	0.954			0.811	0.678	–3.4E-05	0.947	0.091	**0.823**						
D1	0.009	0.881	0.098	0.725					0.110	0.746						
D2	0.005	0.896	0.035	0.543					0.088	0.746	0.109	0.744				
D3	0.003	0.969			1.019	0.597			0.055	0.793						
D4	0.002	0.969			0.972	0.689			0.057	0.800			0.359	**0.747**		
D5	0.010	0.912	0.078	0.780	0.439	0.633			0.122	0.726						
D6	0.006	0.935	0.037	0.623	0.622	0.612			0.101	0.755	0.144	**0.758**				
D7	0.006	0.927	0.034	0.792	0.663	**0.762**			0.098	0.746	0.133	0.552	0.175	0.601		
D8	0.008	0.910	0.038	0.651	0.719	0.641	0.005	0.380	0.110	0.734			0.193	0.537		
D9	0.006	0.942	0.027	0.785	0.524	0.704	0.403	0.480	0.099	0.751	0.136	0.456	0.259	0.374		
D10	0.006	0.946	0.027	**0.797**	0.539	0.646	0.520	0.483	0.098	0.718	0.114	0.441	0.265	0.293	0.189	0.368
D11	0.004	0.702			–0.004	0.702			0.089	0.583			0.228	0.647		
D12	0.005	0.666	0.053	0.700	0.002	0.430			0.097	0.741	0.072	0.353	0.322	0.562		
D13	0.008	0.642	0.083	0.607	0.004	0.536	0.226	0.423	0.103	0.686			0.306	0.502		
D14	0.006	0.646	0.052	0.536	0.002	0.504	0.510	0.303	0.097	0.663	0.075	0.317	0.333	0.489		
D15	0.006	0.726	0.046	0.547	0.002	0.584	0.607	0.660	0.100	0.767	0.054	0.405	0.324	0.543	0.221	**0.480**



Only one parameter listed per column is optimized in a given model. For example, the PID models have an αP parameter whereas the DIVA models have an αA parameter. See Table 1 for a complete list of parameters included in each model.

ICC, intraclass correlation coefficient.

Boldface type indicates the model with the highest ICC value per parameter.

Light blue shading indicates ICC values are between 0.5 and 0.75 (moderate reliability).

Dark blue shading indicates ICC values are > 0.75 (good-excellent reliability).

Generally speaking, parameter stability was higher for the PID-based models and models D1–D10 compared to models D11–D15. In particular, all PID models and models D1–D9 had highly reliable values for the auditory feedback control gain parameter (α_*P*_ in PID models and α_*A*_ in DIVA-based models) and moderately to highly reliable values for the auditory feedback control delay parameter in both studies. The somatosensory feedback control gain parameter was also moderately to highly reliable in all DIVA-based models (D1–D15) in both studies, and the parameter α_*I*_ was highly reliable in the PI and PID models in both studies.

## Discussion

The primary goal of this study is the identification of a model that captures population responses in auditory perturbation experiments, and perhaps more importantly characterizes individual differences in a stable manner with parameters that relate to underlying motor control capabilities. The latter capability is particularly important if the model is to be used to characterize individuals with communication disorders for the purpose of providing individualized treatments that capitalize on the individual’s strengths and weaknesses. For this approach to bear fruit, it is important that the behavioral responses exhibited by experimental subjects are reasonably stable and differ between individuals; if not, then no model will be capable of achieving our goal. A key finding from the current study (independent of any modeling) is that reflexive responses to *f*_*o*_ perturbations are largely individual-specific, providing optimism that such responses may reveal key insights into the individual’s speech motor control processes. Although all subjects were healthy adults with no communication disorders (and therefore likely to have somewhat similar speech motor systems, in contrast to individuals with a speech disorder), the cross-validation classification analyses indicate that the mean of 10 reflexive responses from an individual is enough to distinguish that individual from another neurotypical individual with approximately 90% accuracy in Study 1 (see *pair* column in *Study 1 Xval Classification* section of Table 2) and 75% in Study 21 (see *pair* column in *Study 2 Xval Classification* section of Table 2). This highlights a rather remarkable property of the PSR independent of any modeling: an individual’s pitch shift response is akin to a “fingerprint” that largely distinguishes them from other individuals (though not to the degree of an actual fingerprint). We expect that individuals with speech motor disorders will show much greater variability than our current healthy sample and therefore may be easier to distinguish based on their reflexive responses; verification of this expectation is an important topic for future research.

The three-parameter model D1 provided fits to group mean data with the lowest cAIC values of any model for both Study 1 and Study 2. Furthermore, this model was within the cAIC threshold of the lowest cAIC for individual subject fits and cross-validation simulations for both studies. Other models that fell into the best model category (i.e., those within the cAIC threshold of the lowest cAIC value) for all simulations were PI, D2, D5, D6, D7, D11, D12, and D13. Model D1 also had amongst the most stable parameters across cross-validation iterations as measured by ICC (see Table 3), and its pairwise classification scores were within 1–2% of the best-performing model.

Concerning the three-parameter model PI, although this model performed well according to the cAIC, parameter stability, and cross-validated classification criteria, the overall shape of the responses of the PI model differed considerably from the shape of the subject responses (compare the fits of models PI and D1 in Figures 2B, 3B). The anomalous response shape for the PI model is the result of the fact that the optimized values for the parameter α_*I*_, which determines how much the corrective response increases as error accumulates, were negative (see Tables 3, 4), indicating that the correction actually *decreased* with accumulating error. This is contrary to the theoretical motivation for this term (which is to increase the correction if the error keeps accumulating) and results in the “inverted U” shape of the PI model responses in Figures 2, 3 that is not found in the data traces nor in model D1. It is also worth noting that fixing α_*I*_ at 0 so it will not go negative reduces the PI and PID models to the poorly performing P and PD models, respectively.

For these reasons, we conclude that the best 3-parameter model for characterizing reflexive responses to *f*_*o*_ perturbations is D1 (EQ5), which has free parameters α_*A*_, τ_*A*_, and α_*S*_. These parameters have straightforward interpretations: α_*A*_ (which corresponds to the parameter B in a state-space formulation—see *Basic DIVA/SS equation* in the *“Materials and Methods”* section) is the gain of the auditory feedback controller’s response to a perceived error, τ_*A*_ is the delay of this response, and α_*S*_ is the gain of the “resistance” to this correction. Within the DIVA model, this latter parameter corresponds to the gain of the somatosensory feedback controller, which is attempting to keep *f*_*o*_ (as detected through somatic sensation, which is not perturbed in the current experiment) at the target level. α_*S*_ is related to the parameter A in a state-space formulation (specifically, A = – α_*S*_); this parameter similarly acts to resist changes due to perceived auditory error, though it is not typically specifically associated with somatosensory feedback control. Model D1 is also equivalent to a low-pass filter/leaky integrator model, as proposed by Larson et al. (2000).

A more general interpretation of α_*S*_, which is consistent with both the DIVA and state-space formulations is that it reflects the influence of non-auditory-based motor subsystems on the overall motor output. This can include both feedforward control mechanisms and somatosensory feedback control mechanisms. Indeed, the estimate of the somatosensory state in DIVA is envisioned as a combination of an efference copy of the motor command (which provides a predictive estimate of somatosensory state) and incoming somatosensory information (see for example Figure 1 in Guenther et al., 1998). The use of a predictive estimate of the sensory state within a sensory feedback control architecture (see also Houde and Nagarajan, 2011) is, in essence, a form of feedforward control since it does not depend on sensory feedback for generating control signals.

**FIGURE 4:**
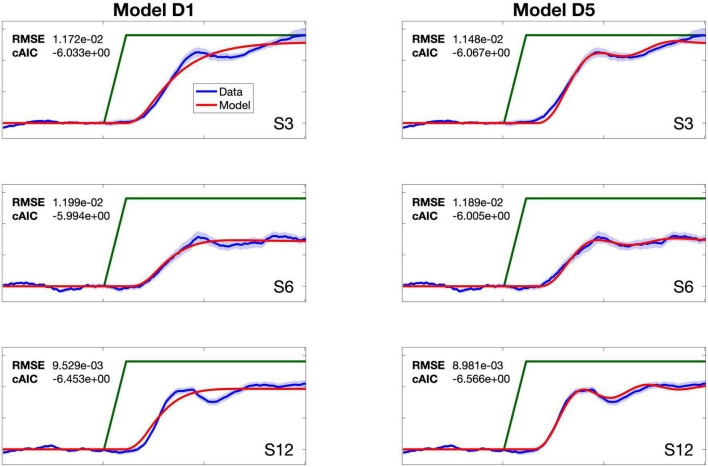
Comparison of the fits for models D1 and D5 for three subjects (S3, S6, S12) from Study 1 who showed multi-component response profiles. Subjects’ mean data traces and standard error of the mean are shown with blue line and shading, respectively. Model fits shown with red line.

Amongst the four-parameter models (PID, D2, D4, D5, and D11), models D5 and D11 were within the cAIC threshold of the lowest cAIC for all simulations for both studies (shaded cells in Table 2), and both of these models exhibited relatively high parameter stability (Table 3). Of these two models, D5 produced fits that better captured the overall shapes of the response profiles (Figures 2, 3). Although the cAIC values for models D5 and D11 were in no cases significantly better than the 3-parameter model D1, it is noteworthy that models D5 and D11 (as well as most of the models with five or more parameters) are better capable of accounting for multi-component response profiles. This is illustrated in Figure 4, which compares the fits of models D1 and D5 to individual subjects from Study 1 who exhibited multi-component responses. Multi-component responses have also been reported in several prior PSR studies (Burnett et al., 1997, 1998; Larson, 1998; Larson et al., 2000; Hain et al., 2000), and it appears that the second response component is under more conscious control than the earlier “automatic” component; for example, the second component is much more influenced by instructions provided to subjects regarding whether they should attempt to oppose or follow the perturbation direction (Hain et al., 2000). The 4th parameter in model D5 is an auditory velocity error gain, α_*AV*_. This term has the effect of resisting any perceived changes in pitch (beyond the abrupt change at perturbation onset, which is ignored by the model), in keeping with the fact that subjects are attempting to maintain a constant pitch, as they were instructed to do in the studies modeled here.

Despite D5 better capturing multicomponent responses, D5 was not superior to D1 according to the cAIC criterion in any of the simulations; in other words, the reduction in RMSE afforded by the 4th parameter in D5 was offset by the AIC penalty term for increasing the number of model parameters by 1. This suggests that the secondary responses, which are better characterized by D5, are quite variable compared to the primary response, which is captured well by both D1 and D5. While the later components could be more influenced by cognitive variables such as attention level and conscious intent (Burnett et al., 1997, 1998; Hain et al., 2000), modeling the contribution of those processes was beyond the scope of the current study. Additional parameters beyond 4 provide little additional improvement.

It is reasonable to wonder what is gained from characterizing and individual’s reflexive responses to *f*_*o*_ perturbations with a parameterized model, given that the average of a set of training traces provides classification results that are on par with the best model characterizations. The key difference is that *a model whose parameters correspond to physiological motor control processes provides a quantitative assessment of an individual’s motor speech capabilities.* For example, a past pitch perturbation study involving individuals with Parkinson’s disease indicated greater compensation than age-matched controls (Liu et al., 2012). By itself, this observation is of limited value for characterizing the motor control processes of an individual with Parkinson’s disease since a larger response might indicate enhanced auditory feedback control or, alternatively, degraded somatosensory feedback control. In contrast, the optimal fit of model D1 to the subject’s response traces provides values of α_*A*_ and α_*S*_ that best capture the subject’s response. These values can be compared to normative values to separately assess the integrity of the auditory and somatosensory feedback control subsystems. If, for example, an individual with Parkinson’s disease has an abnormally low α_*S*_ with normal α_*A*_, a clinician may favor approaches that leverage intact auditory feedback control capabilities to overcome deficient somatosensory feedback control capabilities. In contrast, the parameters in the PID models are interpreted relative to error correction (and whether that correction is proportional to the error, or an integral or derivative of the error). This interpretation does not convey information about the mechanisms driving the correction and may limit how that information could be used in a therapeutic context. Although much work remains to be done to verify the veracity of the D1 model’s characterization, such an approach holds the promise of informing personalized therapeutic interventions, much like other reflexes such as the pupillary light reflex have proven useful for characterizing the integrity of the nervous system in cases of neurological impairment.

## Data availability statement

The raw data supporting the conclusions of this article will be made available by the authors, without undue reservation.

## Author contributions

EK and FG conceptualized and designed the study. FG and AD developed the computational models. EK, AN-C, and RF developed the software. EH and DS collected and processed the data. EK, AN-C, and FG wrote the first draft of the manuscript. All authors contributed to manuscript revision, read, and approved the final submitted version.

## Footnotes

1 We use continuous time notation here for clarity, although the simulations utilize a discrete time representation with one time point per data sample. The data modeled here were sampled at 200 Hz; accordingly, the simulations utilize 5 ms time steps.

2 This model is also equivalent to the SS model of sensorimotor adaptation posed by Daliri and Dittman (2019) with the parameter *a* from that model equal to 1 - α_*S*_, parameter *b* equal to α_*A*_, and removal of τ_*A*_ from the current model since sensorimotor adaptation data were modeled on a trial-by-trial basis rather than a timepoint-by-timepoint basis by Daliri and Ditman. The model is also equivalent to a leaky integrator of the error signal with the leak rate parameter equal to α_*S*_.

3 For example, it does not make much sense within the DIVA model for the auditory feedback gain to be less than 0 (which would exacerbate rather than correct auditory errors) or greater than 1 (which would overcompensate for auditory errors). The bounds used here are slightly larger than these to allow for random variation that may occur in any particular dataset.

4 We chose AIC here over the closely related Bayesian Information Criterion (BIC) because we anticipate that the model training datasets will generally be small; in such cases BIC tends to choose models that are too simple due to its use of a stronger penalty term for the number of model parameters (Burnham and Anderson, 2002).
