# Supplementary material for: Quantitatively characterizing reflexive responses to pitch perturbations
Source: Front Hum Neurosci. 2022 Nov 2;16:929687. doi: 10.3389/fnhum.2022.929687 (PMC9666385; doi:10.3389/fnhum.2022.929687)
Supplement: Supplementary file 1 [file Data_Sheet_1.PDF]

Supplementary Material

1 Fits to group means (all models)

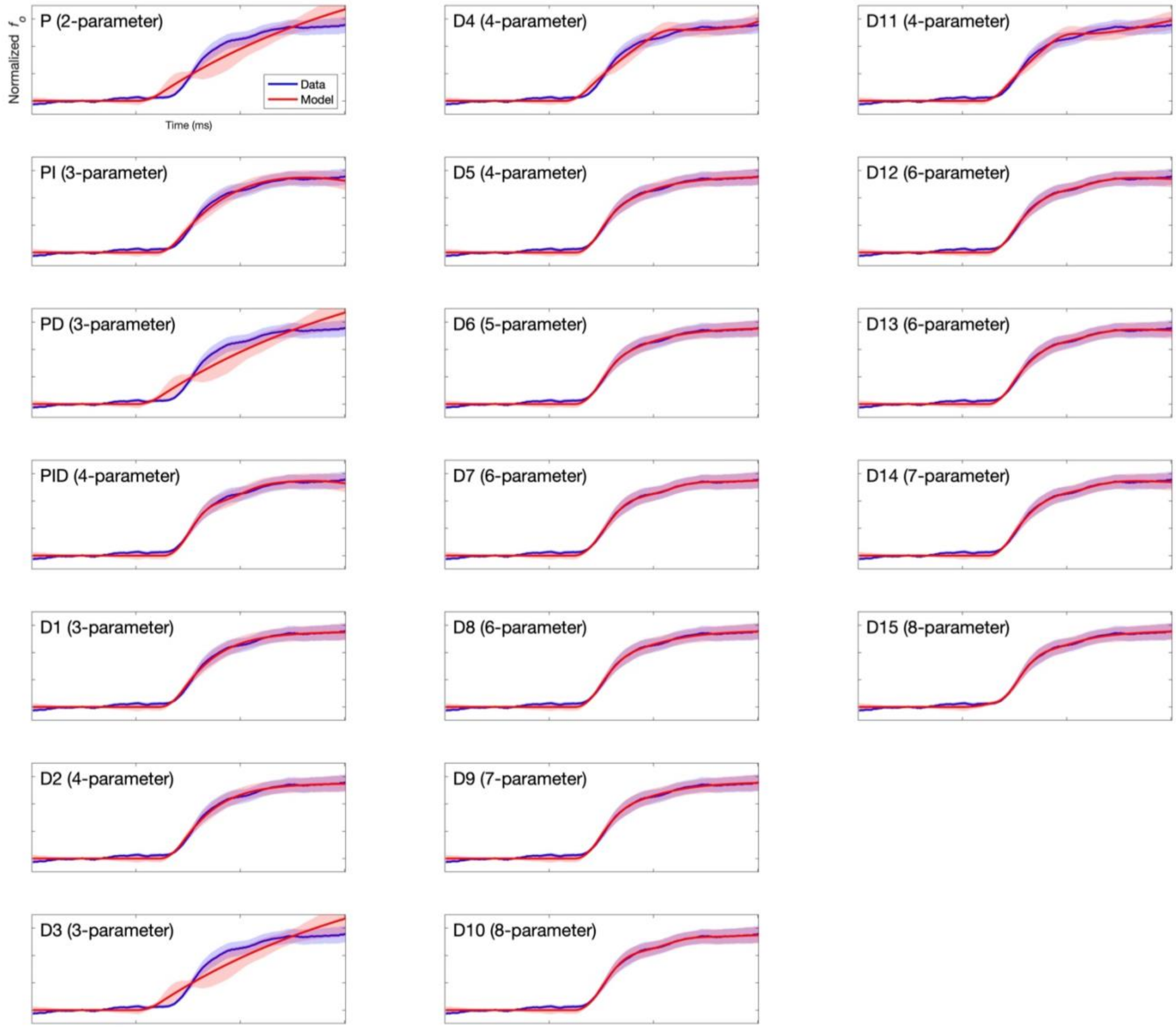

Supplementary Figure 1. Group mean data and model fits for Study 1. Group mean data and standard error of the mean are shown with blue line and shading. Model fit and standard error of the model fit are shown with red line and shading.

.

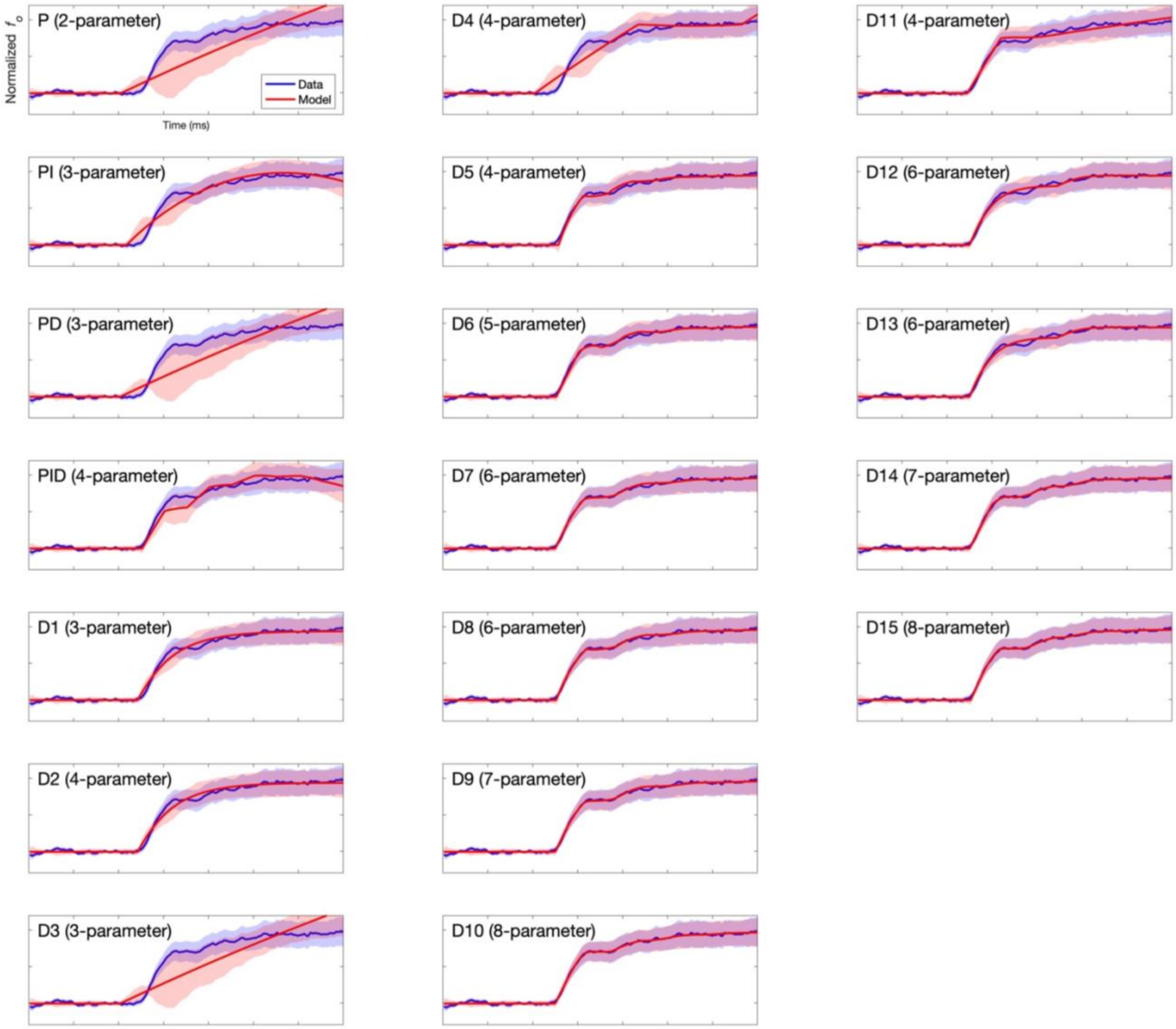

Supplementary Figure 2. Group mean data and model fits for Study 2. Group mean data and standard error of the mean are shown with blue line and shading. Model fit and standard error of the model fit are shown with red line and shading.

2 Optimized parameter values for fits to group means (all models)

	α_P/α_A		α_S		$\alpha_D/\alpha_{Av}/\alpha_{As}$		$\alpha_I/\alpha_{Sv}/\alpha_{Ss}$		τ_A		τ_S		τ_{Av}/τ_{As}		τ_{Sv}/τ_{Ss}	
Model	Study 1	Study 2	Study 1	Study 2	Study 1	Study 2	Study 1	Study 2	Study 1	Study 2	Study 1	Study 2	Study 1	Study 2	Study 1	Study 2
P	0.005	0.001							0.000	0.002						
PI	0.009	0.003					-5.0E-05	-2.0E-05	0.096	0.030						
PD	0.009	0.003			1.056	1.050			0.001	0.002						
PID	0.012	0.005			0.447	0.746	-6.0E-05	-3.0E-05	0.130	0.100						
D1	0.011	0.005	0.011	0.025					0.111	0.080						
D2	0.010	0.005	0.011	0.025					0.111	0.081	0.035	0.011				
D3	0.009	0.003			1.056	1.050			0.001	0.002						
D4	0.007	0.002			0.647	0.973			0.070	0.005			0.355	0.455		
D5	0.012	0.008	0.013	0.040	0.189	0.486			0.125	0.109						
D6	0.010	0.006	0.011	0.027	0.271	0.594			0.120	0.100	0.120	0.100				
D7	0.009	0.006	0.010	0.027	0.527	0.352			0.114	0.095	0.300	0.039	0.066	0.035		
D8	0.021	0.008	0.022	0.039	0.296	0.483	0.800	0.136	0.125	0.100			0.041	0.035		
D9	0.010	0.006	0.011	0.027	0.138	0.347	-0.098	-0.095	0.122	0.096	0.017	0.025	0.037	0.033		
D10	0.010	0.006	0.011	0.026	-0.049	0.089	0.484	0.362	0.121	0.096	0.234	0.055	0.183	0.194	-0.072	0.069
D11	0.008	0.005			-0.005	-0.004			0.091	0.085			0.329	0.150		
D12	0.010	0.006	0.014	0.038	0.002	0.001			0.114	0.094	0.064	0.019	0.375	0.391		
D13	0.011	0.006	0.014	0.038	0.002	0.001	-0.100	-0.100	0.120	0.094			0.376	0.391		
D14	0.011	0.005	0.015	0.029	0.002	0.001	-0.048	0.483	0.122	0.096	0.019	0.101	0.375	0.420		
D15	0.002	0.006	0.015	0.032	0.012	0.001	0.316	0.296	0.005	0.100	0.004	0.040	0.146	0.416	0.137	0.084

Note. Only one parameter listed per column is optimized in a given model. For example, the PID models have an α_P parameter whereas the DIVA models have an α_A parameter. See Table 1 for a complete list of parameters included in each model.
